# Transmission of and Infection With COVID-19 Among Vaccinated and Unvaccinated Attendees of an Indoor Wedding Reception in Minnesota

**DOI:** 10.1001/jamanetworkopen.2022.0536

**Published:** 2022-02-25

**Authors:** Haley Wienkes, Kelley Vilen, Alexandra Lorentz, Daniel Gerlach, Xiong Wang, Amy Saupe, Richard Danila, Ruth Lynfield, Kirk Smith, Carlota Medus

**Affiliations:** 1 Minnesota Department of Health, St Paul; 2 Association of Public Health Laboratories, Silver Spring, Maryland

## Abstract

**Importance:**

Characterizing rates of SARS-CoV-2 infection among vaccinated and unvaccinated persons with the same exposure is critical to understanding the association of vaccination with the risk of infection with the Delta variant. Additionally, evidence of Delta variant transmission by children to vaccinated adults has important public health implications.

**Objective:**

To characterize transmission and infection of SARS-CoV-2 among vaccinated and unvaccinated attendees of an indoor wedding reception.

**Design, Setting, and Participants:**

This cohort study included attendees at an indoor wedding reception in Minnesota in July 2021. Data were collected from REDCap surveys and routine surveillance interviews. The full list of attendees and a partial list of emails were obtained. Fifty-seven attendees completed the emailed survey. Eighteen additional attendees were identified from the state health department COVID-19 surveillance database.

**Exposures:**

Attendance at an indoor event.

**Main Outcomes and Measures:**

Risk of SARS-CoV-2 infection among vaccinated and unvaccinated attendees, identification of an index case, whole genome sequencing (WGS) to identify the COVID-19 variant, understanding of transmission patterns, and assessment of secondary transmission. The primary case definition was an individual with a positive SARS-CoV-2 test who attended the wedding in the 14 days prior to their illness.

**Results:**

Data were gathered for 75 attendees (mean [SE] age, 37.5 [13.7] years; 57 [76%] female individuals), of whom 56 (75%) were fully vaccinated, 4 (5%) were partially vaccinated, and 15 (20%) were unvaccinated. Of 62 attendees who were tested, 29 (47%) tested positive, including 16 of 46 fully vaccinated attendees (35%), 2 of 4 partially vaccinated attendees (50%), and 11 of 12 unvaccinated attendees (92%). Being unvaccinated was associated with a higher risk of infection compared with being vaccinated (risk ratio, 2.64; 95% CI, 1.71-4.06; *P* = .001). One unvaccinated adult required hospitalization. An unvaccinated child who was symptomatic on the event date was identified as the index case. Eleven specimens were available for WGS. All sequenced specimens were closely related and were identified as the Delta variant. WGS supported secondary transmission from a vaccinated individual with SARS-CoV-2.

**Conclusions and Relevance:**

This cohort study identified a COVID-19 Delta variant outbreak at an indoor event despite a high proportion of vaccinated attendees. It found that vaccination was associated with a reduced risk of infection.

## Key Points

**Question:**

What are the characteristics of SARS-CoV-2 infection and transmission among vaccinated and unvaccinated attendees of a large indoor gathering?

**Findings:**

In this cohort study of 75 individuals, nearly half of attendees at an indoor wedding reception who were tested were infected with the Delta variant of SARS-CoV-2. Unvaccinated attendees had a higher risk of SARS-CoV-2 infection than vaccinated attendees, secondary transmission from vaccinated attendees to vaccinated and unvaccinated contacts was observed, and the index case was identified as an unvaccinated symptomatic child.

**Meaning:**

These findings suggest that unvaccinated people are at increased risk of contracting SARS-CoV-2 compared with vaccinated people in large social gatherings.

## Teaser

This cohort study characterizes transmission and infection of SARS-CoV-2 among vaccinated and unvaccinated attendees of an indoor wedding reception.

## Introduction

Social gatherings, such as weddings, have been associated with increased risk of transmission of SARS-CoV-2, the virus that causes COVID-19.^1,2^ The Delta variant (first identified in December 2020) of SARS-CoV-2 is a variant of concern and has been shown to have increased transmissibility and an increased ability to infect vaccinated individuals.^3^ In July 2021, the Minnesota Department of Health (MDH) investigated an outbreak of COVID-19 at a wedding reception with a high percentage of vaccine breakthrough cases. No masking or social distancing measures were used at the reception. At the time of this outbreak, the US Centers for Disease Control and Prevention (CDC) had lifted its masking recommendation for vaccinated individuals, and Minnesota had fully vaccinated 52.2% of the overall population.^4^ This cohort study summarizes the findings of the outbreak investigation.

## Methods

This was an epidemiologic assessment of an outbreak investigation using a cohort design conducted as part of routine public heath practice activities by the authors at the MDH. Under Minnesota reporting rule 4605.7050,^5^ health care practitioners and laboratories are mandated to report information about all persons tested for COVID-19, including but not limited to name, contact information, test date, test results, and details about the illness including onset date if it is known. Persons who test positive for COVID-19 are interviewed by MDH with a questionnaire about illness and exposures. The exposure section includes questions about travel, events, and exposure to a known COVID-19 case prior to their infection. Interview data are reviewed to identify common exposures indicative of a common-source outbreak. Outbreak investigations are considered standard public health practice under Minnesota Statute 144.05,^6^ not research as defined by 45 CFR 46.102(d),^7^ and thus this investigation was not subject to review by an institutional review board.^6,7^

Investigators provided a Tennessen warning^8^ as a form of informed consent for data collected by the state about individuals. Persons can agree or refuse to participate with no consequences. To protect participants’ privacy in this report, the exact number of wedding attendees and some specific details will not be reported. This study followed Strengthening the Reporting of Observational Studies in Epidemiology (STROBE) reporting guideline.

The outbreak was identified through routine COVID-19 case surveillance interviews. The full list of all attendees in the cohort and a partial list of email addresses for 74 participants were obtained. A REDCap survey was sent to all 74 available email addresses in an attempt to capture the complete cohort. The survey included questions about demographic characteristics, symptoms, testing, vaccination, specific wedding event exposures, and close contacts. Additional data, including but not limited to symptom status, test result, onset date, and vaccination status, were gathered from case surveillance interviews and laboratory reports in the Minnesota Electronic Disease Surveillance System (MEDSS) and from immunizations reported to the Minnesota Immunization Information Connection (MIIC). Data from these additional sources were used to verify key data points from survey responses, such as test results and vaccination status, and to supplement missing data for attendees with incomplete or missing survey responses.

A primary case was defined as an individual with a positive SARS-CoV-2 polymerase chain reaction or antigen test that reported attending 1 or more events associated with the outbreak wedding in the 14 days prior to their illness onset date. Test date was used as a proxy for onset date for individuals who did not respond to the survey or interview. Individuals who self-reported a positive test on the REDCap survey but were not confirmed in MEDSS were also considered primary cases. Secondary cases were identified in MEDSS by matching address and telephone numbers of primary cases and by cross-referencing name, telephone number, and date of birth of close contact information provided by primary cases.

Vaccination status was obtained from the REDCap survey or MIIC. Fully vaccinated was defined as receipt of a full series of any COVID-19 vaccine at least 14 days prior to the wedding. Partially vaccinated was defined as receipt of an incomplete series (ie, only 1 dose of the Pfizer or Moderna vaccines) or completion of any series within 14 days of the wedding.

Specimens from testing laboratories were routinely sequenced for variant surveillance. Genomic sequences were requested from the testing laboratories. Additional specimens were requested from clinical laboratories to be sent to the MDH Public Health Laboratory for whole genome sequencing (WGS). WGS was conducted based on modified ARTIC protocol using the version 3 primer set.^9^ Whole genome consensus sequences were generated using containerized Monroe pipeline. The SARS-CoV-2 lineages were identified using pangolin (pangolin version 3.1.16, pangoLEARN 2021-11-18, and pango-designation version 1.2.102).^10,11^ Initial phylogenetic relationships were identified via Nextstrain; Ultrafast Sample Placement on Existing Trees (UShER) was used to identify similar sequences; and Interactive Tree of Life (iTOL) version 6.3 was used to make the final visualization.^12,13,14^

### Statistical Analysis

Statistical analysis was performed using SAS version 9.4. The Fisher exact test was used to calculate the risk ratio (RR) of testing positive between vaccinated and unvaccinated attendees and to determine statistical significance. A 2-sided *P* < .05 was considered statistically significant, and 95% CIs were calculated.

## Results

Eight days after the wedding reception, the event was identified as a common exposure among several individuals with COVID-19. Multiple individuals with positive test results reported attending the wedding when interviewed as part of routine surveillance, and an outbreak investigation was initiated. There were between 100 and 125 attendees at the indoor reception. Overall, 57 surveys were completed, and data were collected from laboratory reports or surveillance case interviews for an additional 18 attendees. Data from a total of 75 persons (mostly wedding attendees and a few event venue employees) were included in the analysis. The mean (SE) age was 37.5 (13.7) years; 57 (76%) were female individuals, and 17 (23%) were male individuals.

The wedding took place in July 2021. There were several wedding events, including a rehearsal dinner, an outdoor ceremony, an indoor reception, and an after-party. Relatively few attended the dinner, ceremony, and after-party. All 75 persons included in this analysis attended the wedding reception, with 34 (including 10 who later tested positive) reporting only attending the reception.

Sixty-two attendees were tested after the wedding. Attendees were tested a median of 5 days after the event (range, 1-30 days). Twenty-nine attendees tested positive (47% of the 62 that were tested, 39% of all 75 respondents), including a small number of event staff from the reception venue. The attack rate was not calculated because data were not available for the entire cohort. However, the minimum possible attack rate if all nonrespondents were not infected would be 25%, and the maximum possible attack rate if all nonrespondents were infected would be 70%.

The 29 individuals with positive results represented 22 households. Two of 29 (7%) self-reported a positive test on the survey but were not verified in MEDSS owing to out-of-state residence or an at-home test; they were included as primary cases. No respondents reported an onset prior to the event; 1 respondent (3%) had a symptom onset on the day of the wedding reception. Although this individual was not interviewed, the test result, date of onset, and symptom status were reported to MDH by the health care practitioner. This was the individual with the earliest onset among the attendees and therefore determined to be the index case. The index case was an unvaccinated child and was excluded from statistical analysis. The source of the index case’s infection was unknown. No individuals reported exposure to a known COVID-19 case outside of other wedding attendees.

Vaccination status was available for all 75 respondents, 56 of whom (75%) were fully vaccinated. Among the 62 respondents who were tested, 16 of 46 fully vaccinated attendees (35%) tested positive as well as 2 of 4 partially vaccinated attendees (50%) and 11 of 12 unvaccinated attendees (92%) (Table). The risk of an unvaccinated attendee being infected with SARS-CoV-2 was significantly higher compared with fully vaccinated wedding attendees (RR, 2.64; 95% CI, 1.71-4.06; *P* = .001). The difference in proportion of individuals testing positive by manufacturer was not statistically significant.

**Table.** Test Results of Wedding Reception Attendees by Vaccination Status

Outcome	Attendees, No. (%)		
	Fully vaccinated (n = 56)	Partially vaccinated (n = 4)	Unvaccinated (n = 14)^a^
Tested (n = 62)	46 (82)	4 (100)	12 (87)
Positive result (n = 29)	16 (35)	2 (50)	11 (92)
Negative results (n = 33)	30 (65)	2 (50)	1 (8)

^a^ Two individuals in this group were children and therefore ineligible for vaccination.

**Figure 1.**
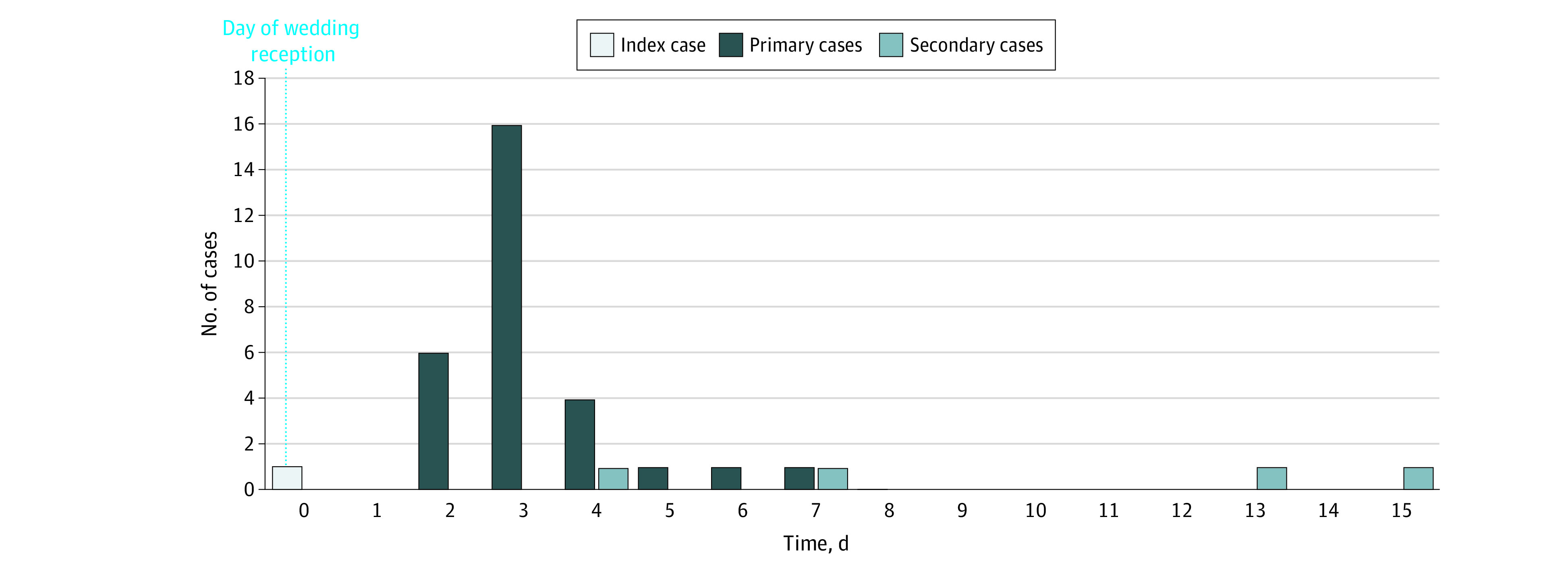
Epidemic Curve of Onset Dates of Index, Primary, and Secondary Cases

Of the 29 individuals who tested positive, 28 (97%) were symptomatic, and for 1 (3%), symptom status was unknown. Test date was used as a proxy for symptom onset date for the individual with unknown symptom status. All symptomatic respondents sought testing and tested positive. The median incubation period was 3 days (range, 2-7 days) (Figure 1).

Specific symptom profile data were available for 23 individuals. The median number of symptoms among fully vaccinated attendees was 9 (range, 5-13), and among unvaccinated attendees, it was 8 (range, 4-13). The median duration of illness was not significantly different between vaccinated individuals (8 days) and unvaccinated individuals (9 days). Only 1 partially vaccinated individual with COVID-19 infection had symptom profile data available; this individual reported experiencing 7 symptoms and an illness duration of 9 days.

An unvaccinated adult younger than 65 years with no underlying health conditions was hospitalized. Overall, 21 contacts were reported by primary cases, 14 of whom were Minnesota residents. Four individuals were identified as secondary cases. Three of the 4 secondary cases (75%) reported being symptomatic, and the fourth secondary case was lost to follow-up. One secondary case attended the wedding but did not test positive until day 13. Given that this secondary case lived with a primary case, their infection was most likely acquired through household exposure. Another individual identified as a secondary case attended the wedding, lives with a primary case, and became symptomatic on day 15. The remaining 2 were social contacts who spent time with a primary case 1 to 3 days after the wedding and became symptomatic 4 and 7 days after the wedding, respectively. Among the secondary cases, 3 (75%) were fully or partially vaccinated and 1 (25%) was unvaccinated. All 4 primary cases that resulted in identified secondary cases were fully vaccinated.

**Figure 2.**
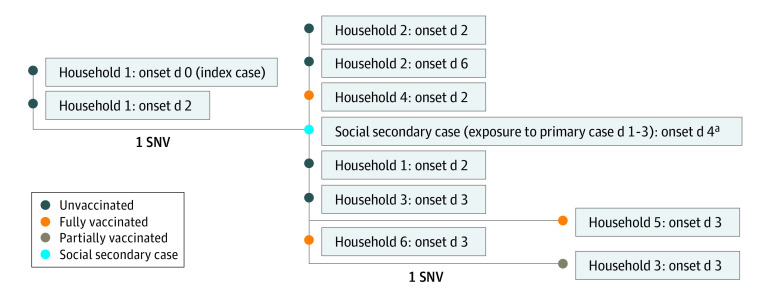
Whole Genome Sequencing Results With Onset as Days Since Wedding All sequences were the Delta variant. Branches are labeled by single-nucleotide variant (SNV) differences. Interactive Tree of Life version 6.3 was used to make this visualization. ^a^Specific exposure date was unknown but was identified to be between day 1 and 3 after the wedding.

Eleven specimens were available for WGS. Sequenced specimens were identified as the AY.39 lineage of the Delta variant.^15^ One additional sample was sequenced and identified as the Delta variant, but it was of insufficient quality to place on the phylogenetic tree. All sequences were within 0 to 2 single-nucleotide variants (SNVs). No other similar sequences in the community were identified prior to the event. The sequenced cases came from 7 households. The index case’s sequence was identical to 1 other case and was 1 to 2 SNVs away from the remaining cases, supporting that this was the likely source of the outbreak. Only 1 secondary case specimen was available for sequencing, and it matched other primary cases (Figure 2).

## Discussion

This cohort study examined a COVID-19 outbreak among attendees of an indoor wedding reception without social distancing or masking. At the time of the event, the 14-day case rate in the affected county was 4.3 cases per 10 000 residents per day.^16^ No attendees reported exposure to a known COVID-19 case outside of the wedding. The likely source was an unvaccinated child with onset on the day of the wedding. WGS data found all sequenced cases to be within 0 to 2 SNVs of each other. Based on previous research, there is high confidence that sequences within 0 to 2 SNVs are part of the same cluster or outbreak.^17,18^ The WGS results supported evidence of transmission from the index case to the remaining individuals and from a primary case to a social secondary case.

This investigation found that outbreaks are possible in highly vaccinated settings. The vaccination rate among respondents (75%) was higher than Minnesota’s statewide vaccination rate (52%)^4^ at the time of the event. Population-level data indicate vaccine breakthrough cases are rare, but there is limited existing literature evaluating the proportion of infected cases in a highly vaccinated cohort with a discrete exposure. These data provide an example of the risk of infection for vaccinated and unvaccinated persons in a highly vaccinated cohort exposed to the Delta variant.

The findings also illustrate the potential for children to spread the Delta variant to vaccinated and unvaccinated populations. Children younger than 12 years were not yet eligible for vaccination at the time of this outbreak, so these findings may have significant implications for mitigation strategies in settings that include children.^19^

Evidence of secondary transmission of SARS-CoV-2 from vaccinated individuals to others was observed in this investigation and supported by WGS results. The frequency of transmission from vaccinated individuals could not be assessed owing to limitations in secondary case finding, but it is an important consideration for testing, isolation, and quarantine recommendations of vaccinated individuals. These data support CDC guidelines suggesting vaccinated individuals adhere to the same isolation protocols as unvaccinated individuals after a COVID-19 diagnosis.^19^

Vaccination reduces the risk of contracting COVID-19.^20^ Fully vaccinated attendees had a lower risk of developing symptoms or testing positive, and the only hospitalization was among an unvaccinated adult, supporting previous data showing reduced risk of infection and severe illness among vaccinated individuals.^21^ Children and those who are unable to be vaccinated should continue to practice masking and social distancing and consider limiting their exposure to large events. Parents of children in age groups eligible for vaccination should consider getting them vaccinated. In addition, the CDC recommends that when community transmission rates are substantial or high, even vaccinated individuals should mask indoors, and some vaccinated individuals may choose to wear a mask at other times, particularly if they have or live with someone who has immunocompromise or underlying conditions. The CDC recommendation for vaccinated individuals to mask while indoors during times of substantial community transmission was not in place at the time of this wedding.

Additionally, this investigation found transmission to event employees, showing that employees are at risk from the patrons they serve in indoor gatherings. Businesses that host large events should take these risks into consideration when determining which mitigation measures to put into place.

In this investigation, WGS was used to confirm transmission of SARS-CoV-2. Routine sequencing is a powerful tool to aid in the understanding of transmission patterns.

### Limitations

This study has several limitations. The sample size was small. Email addresses were only obtained for 74 guests; therefore, there is possible sampling bias. Nonresponse bias may also have been present, as only 57 of the 74 surveys were completed. Where available, information about persons who did not respond to the REDCap survey were collected from surveillance case interviews and test results, but there were still remaining attendees for whom no data were obtained; therefore, they were excluded from the denominator. It is possible that persons in this cohort were not tested due to a lack of symptoms, resulting in an overstatement of the true proportion of infected attendees or an understatement of the proportion of asymptomatic cases. Ability to detect secondary cases was limited, so some secondary cases may have been missed. Only 11 samples were available for WGS. Not all secondary cases were sequenced, and therefore chains of transmission were not confirmed by WGS in those cases. There were limited data for the index case, and the index case lived in the same household as other outbreak cases; therefore, it is possible that those in the index case’s household may have been infected at a different point in the index case’s infectious period.

## Conclusions

In this cohort study of an outbreak investigation, a large outbreak of the Delta variant of COVID-19 occurred, even with a high proportion of vaccinated people at the event. This investigation found a high rate of vaccine breakthrough infections with the circulating Delta variant. However, unvaccinated wedding attendees were 2.6 times more likely to be infected with SARS-CoV-2 compared with vaccinated attendees. The only illness severe enough to require hospitalization was in an unvaccinated adult. A symptomatic child was identified as the index case. Multiple mitigation measures may be needed to protect against transmission of highly contagious variants of SARS-CoV-2 in large social gatherings when community rates of transmission are elevated, even when a high proportion of attendees are vaccinated.
